# Magnetic resonance imaging of placentome development in the pregnant Ewe

**DOI:** 10.1016/j.placenta.2021.01.017

**Published:** 2021-01-26

**Authors:** Dimitra Flouri, Jack R.T. Darby, Stacey L. Holman, Sunthara R. Perumal, Anna L. David, Janna L. Morrison, Andrew Melbourne

**Affiliations:** a School of Biomedical Engineering and Imaging Sciences, Kings College London, London, United Kingdom; b Department of Medical Physics and Biomedical Engineering, University College London, London, United Kingdom; c Early Origins of Adult Health Research Group, Health and Biomedical Innovation, UniSA Clinical and Health Sciences, University of South Australia, Adelaide, Australia; d South Australian Health & Medical Research Institute, Preclinical, Imaging & Research Laboratories, Adelaide, Australia; e Elizabeth Garrett Anderson Institute for Women’s Health, University College London, London, London, United Kingdom; f NIHR Biomedical Research Centre, University College London Hospitals, London, United Kingdom

**Keywords:** Diffusion, Placenta, IVIM, Relaxometry, Anisotropy

## Abstract

**Introduction:**

Novel imaging measurements of placental development are difficult to validate due to the invasive nature of gold-standard procedures. Animal studies have been important in validation of magnetic resonance imaging (MRI) measurements in invasive preclinical studies, as they allow for controlled experiments and analysis of multiple time-points during pregnancy. This study characterises the longitudinal diffusion and perfusion properties of sheep placentomes using MRI, measurements that are required for future validation studies.

**Methods:**

Pregnant ewes were anaesthetised for a MRI session on a 3T scanner. Placental MRI was used to classify placentomes morphologically into three types based on their shape and size at two gestational ages. To validate classification accuracy, placentome type derived from MRI data were compared with placentome categorisation results after delivery. Diffusion-Weighted MRI and T2-relaxometry were used to measure a broad range of biophysical properties of the placentomes.

**Results:**

MRI morphological classification results showed consistent gestational age changes in placentome shape, as supported by post-delivery gold standard data. The mean apparent diffusion coefficient was significantly higher at 110 days gestation than at late gestation (~140 days; term, 150 days). Mean T2 was higher at mid gestation (152.2 ± 58.1 ms) compared to late gestation (127.8 ms ± 52.0). Significantly higher perfusion fraction was measured in late gestation placentomes that also had a significantly higher fractional anisotropy when compared to the earlier gestational age.

**Discussion:**

We report baseline measurements of techniques common in placental MRI for the sheep placenta. These measurements are essential to support future validation measurements of placental MRI techniques.

## 1 Introduction

The placenta is critical for normal fetal growth and development. This unique organ is responsible for the exchange of substrates and waste products between the mother and her fetus; and is therefore, the main regulator of fetal growth [1,2]. Abnormalities of placental development and function may result in preeclampsia, placental insufficiency and fetal growth restriction (FGR) [3]. Nevertheless, our knowledge regarding placental growth and function remains limited. Most of the information that we currently have on human placental biology has been obtained by studying placental tissue after delivery or using *ex-vivo* models [4–9]. Understanding the structure and function of the placenta *in-vivo* during gestation will lead to a greater understanding of placental biology and thus the development of both preventative and therapeutic interventions against feto-placental pathology that have a continued impact across the life-course [10].

Magnetic resonance imaging (MRI) is a powerful non-invasive imaging technique that offers superb visualisation of the lobular placental anatomy and allows quantitative analysis of spatial and temporal changes in tissue morphology. Placental MRI has promise for improving prediction, diagnosis and monitoring of pregnancy complications [11, 12]. Several MRI studies of placental anatomy and perfusion have been attempted in different species [13–18]. A unique MRI feature of blood is that the T2, a measure of the rate of excited protons going out of phase, depends on the oxygen saturation of haemoglobin, as well as on water diffusion and exchange between water and other molecules. For a given haematocrit the blood T2 is positively associated with its oxygen saturation due to the paramagnetic effect of deoxyhaemoglobin [19]. T2-relaxometry has identified large effect sizes in human pregnancies complicated by FGR and has potential to estimate oxygenation levels of the fetus [20–22].

Diffusion-Weighted (DW) MRI characterises water motion on a molecular level and provides information on placental cellularity, micro-structure and function [23–25] while diffusion tensor imaging (DTI) can measure the diffusion directionality imposed by the geometrical arrangement of tissue microstructure [26]. Fractional anisotropy (FA) is a DTI measure extracted from diffusion images that represents preferred directionality in water diffusion. A significant reduction of FA has been linked to diminished functional placental tissue [27]. The apparent diffusion coefficient (ADC), linked to the overall cellularity of the tissue, serves as convenient measure of the magnitude of diffusion and shows alterations in placental pathology such as FGR [25]. One variant of DWI, intravoxel incoherent motion model (IVIM) can obtain placental perfusion-related measures by collecting data over a range of diffusion weightings [23,28,29]. This technique measures the volume of blood flowing within the capillaries and is found to have predictive value for late-onset FGR [30].

Validating measurements of flow and oxygenation throughout pregnancy in humans is challenging due to the need to avoid risk to both the mother and fetus. To that end, sheep are particularly useful for studying placental biology [26,31–35], although the anatomical structure of their placenta is not the same as humans [36]. Sheep are amenable to surgical intervention and represent a large animal model of relevant dimensions to human pregnancy and are thus suitable for study in standard clinical imaging machines with standard imaging protocols. Models of placenta mediated FGR also exist in this animal model, making it suitable for studying placental function in pathology [32,37]. In contrast to the human discoid placenta, the sheep placenta is comprised of multiple discrete placentomes [8,38,39]. It is currently unknown if there are different functional roles for the distinct placentome types [38], but it is plausible that any differences seen between placentome types will have an analogous relationship to the more amorphous lobular structure of the human placenta, where functional differences between adjacent lobules are equally poorly understood. Sheep are thus likely to be a relevant model of human placental function at this scale. Nutrient and gas exchange in sheep take place between interdigitated fetal and maternal villi; however, in the human placenta there is no maternal villus structure. Despite these differences, the physiological similarity in function of the fetal placental vascular structure and fetal blood physiology, oxygenation and both maternal and fetal size mean that sheep serve as a useful model for placental vascular development and gas exchange [34,36,40].

We report baseline measurements of placental MRI measures for the sheep placenta across gestation. We characterise the diffusion and perfusion properties of the sheep placentome including the ADC, T2, fractional anisotropy (FA) and perfusion fraction (*f*) derived from IVIM analysis on sheep placental tissue in early and late gestation.

## 2 Materials and methods

### 2.1 Animals

The study and all animal handling procedures were approved by the Animal Ethics Committee of the South Australian Health and Medical Research Institute (SAHMRI) and abide by the Australian Code of Practice for the Care and Use of Animals for Scientific Purposes developed by the National Health and Medical Research Council. Ewes from the SAHMRI farm (Burra, South Australia) were housed in an indoor facility with a constant ambient temperature of 20–22 °C and a 12-h light/dark cycle. Ewes were housed in individual pens in view of other sheep and had *ad libitum* access to food and water. All investigators understood the ethical principles outlined in Grundy et al. [41] and the principles of the 3Rs [42].

Singleton-bearing Merino ewes (n = 14) were selected at 105–110 days gestation (term = 150). All ewes underwent fetal catheterisation surgery at 116–117 days gestation under aseptic conditions as previously described [37,43,44]. General anaesthesia was induced with intravenous diazepam (0.3 mg/kg) and ketamine (7 mg/kg) and then maintained with isoflurane (2.5% in 100% oxygen). Vascular catheters were implanted into the maternal jugular vein, fetal femoral vein and femoral artery as well as the amniotic cavity. The fetus was returned to the uterus. A small incision was made in the ewes’ flank, allowing exteriorisation of the fetal catheters. Ewes received an intramuscular injection of antibiotics 3.5 ml of Duplocillin (150 mg/ml procaine penicillin and 112.5 mg/ml benzathine penicillin; Norbrook Laboratories Ltd., Gisborne, Australia) and 2 ml of 125 mg/ml Dihydros-treptomycin (Sigma, St Louis, MO, USA) for 3 days following surgery. Fetuses received an intramuscular injection of 1 ml of Duplocillin and 1 ml Dihydrostreptomycin during surgery. All ewes received the analgesic meloxicam (0.5 mg/kg, subcutaneously) on the day before surgery and 24 h later [45]. Each fetus received antibiotics (500 mg; sodium ampicillin, Commonwealth Serum Laboratories) intra-amniotically for 4d post-surgery. After fetal surgery, fetal arterial blood samples were collected daily to monitor fetal health by measuring the partial pressure of oxygen (PaO_2_), partial pressure of carbon dioxide (PaCO_2_), oxygen saturation (SaO_2_), pH, haemoglobin, haematocrit, base excess and lactate, calibrated for sheep blood with a RAPIDPOINT 500 (Siemens Healthineers, Erlangen, Germany).

### 2.2 MR imaging

At 109–111 (n = 14) and 139–140 (n = 5) days gestation, ewes underwent MRI scans. General anaesthesia was induced and maintained as per surgery. The ewe was then positioned on its left side for the duration of the scan and ventilated to create normal fetal oxygen levels (respiratory rate 16–18; ~1L O_2_ and 5L air (109–111 d GA) or ~2L O_2_ and 4L air (139–140 d GA) [46]. Maternal heart rate and oxygen saturation were measured using an MRI compatible SaO_2_/heart rate monitor (Nonin Medical Inc, Plymouth, USA). The sensor was placed on the pregnant ewes’ teat and measurements were continuously recorded using LabChart 7 (ADInstruments, Castle Hill, Australia) [21]. MRI was performed on a 3T Siemens Skyra Scanner (Erlangen, Germany). Diffusion imaging was performed at seven b-values = [0, 10, 20, 30, 50, 70, 100, 200, 300, 500, 600] s.mm^−2^ and echo time (TE) = 72 ms. A spin-echo T2 relaxometry acquired at b-value = 0 s.mm^−2^ and at ten TE = [81, 90, 96, 120, 150, 180, 210, 240, 270, 300] ms. In addition, data acquired at b-values 50 and 200 for TE = [81, 90, 120, 150, 180, 210, 240] ms. Voxel size was 0.9 × 0.9 × 2.5 mm^3^, reconstructed matrix 308 × 384 and 26 slices. Diffusion and relaxation measurements were obtained using a pulsed gradient spin-echo with an EPI readout. DTI was acquired in 30 non-colinear directions at b-values 50 s.mm^−2^ and 100 s. mm^−2^ and TE 63 ms and 69 ms respectively. DTI voxel size was 3.6 × 3.6 × 2.5 mm^3^, reconstructed matrix 78 × 96 and 36 slices.

### 2.3 Modelling

#### 2.3.1 Single-tissue compartment model

The simplest model for analysing MR data considers singlecompartment models. The calculation of ADC often assumes a particular model of single-exponential decay given by:

(1) $$S\left(b\right)=S_{0}e^{-bADC},$$

where *S*(*b*) is the signal at a diffusion weighting with b-value b and *S*_0_ is the signal with zero diffusion weighting, i.e. b = 0.

Another single-tissue compartment model is T2-relaxometry. In T2-relaxometry the parameter of interest is the T2 relaxation rate itself and is given by the following monoexponential decay:

(2) $$S\left(TE\right)=S_{0}e^{-TE/T2},$$

where *TE* is the echo time and T2 is the relaxation time.

#### 2.3.2 DTI-model

DTI features diffusion measurements in at least six directions. The acquired diffusion measurements along the six axes are fitted to a 3 × 3 symmetric matrix, the diffusion tensor (**D**). The properties of the diffusion tensor are characterised by the eigenvectors (*v*_1_, *v*_2_, *v*_3_) and the eigenvalues (*λ*_1_, *λ*_2_, *λ*_3_). The FA is frequently used to measure diffusion anisotropy or directionality and ranges from 0 (isotropic: no preferred direction) to 1 (anisotropic: only one direction) [39]. The DTI model as described in Ref. [48] is of the form:

(3) $$S\left(b,r\right)=S_{0}e^{-lr^{T}Dr},$$

where *S*(*b*, ***r***) is the signal at diffusion weighting with b-value b and direction ***r***.

#### 2.3.3 IVIM-model

Initially proposed by Le Bihan et al. [23], IVIM enables simultaneous evaluation of perfusion and diffusion through a multi-b-value DWI. The IVIM model is commonly formulated as a two-compartment model and is given by the biexponential equation:

(4) $$S\left(b\right)=S_{0}\left[fe^{-bxl}+\left(1-f\right)e^{-bADC}\right],$$

where *f* is the perfusion fraction and d* is the pseudo-diffusion coefficient.

### 2.4 Collection and analysis of placentomes

At 139–141 days GA, all pregnant ewes were euthanised with an overdose of sodium pentobarbitone (Virbac, Peakhurst, NSW, Australia) and the fetus was delivered via hysterotomy and weighed. After the fetus was removed, placentomes were dissected from the uterus and classified morphologically [38,47]. This serves as the gold standard against which the MRI technique was compared. Whilst 14 ewes were initially enrolled in the study and received the initial MRI scan at 109–111 d GA, only 5 of these ewes received a paired MRI scan at 139–141 d GA. Four of these ewes had placentome collection and classification on the next day (one ewe delivered between MRI and tissue collection). Post-mortem measurements and number of placentomes observed are provided in Table 1. It was not possible to match placentomes at post-mortem to those captured by MRI. We did attempt to label placentomes at surgery with copper wire, but this was not visible on the MRI images. Regardless, this would not be possible for all placentomes as they could not all be located at surgery or discriminated in MRI images.

### 2.5 Image processing

We manually defined regions of interest (ROIs) containing the placentomes on the first b = 0 image (ITK-SNAP Version 3.6.0, 2017). The ROIs covered a representative area of each placentome, and ROIs were placed away from edges such that any residual movement artefact would not cause it to move out of the placentome. Although data did not suffer from significant motion artefacts, to mitigate motion a non-rigid registration method was used to align all images [49]. For each ewe, the same ROIs were used for all images in the dynamic series.

### 2.6 MRI derived placentome morphological classification

**Fig. 1:**
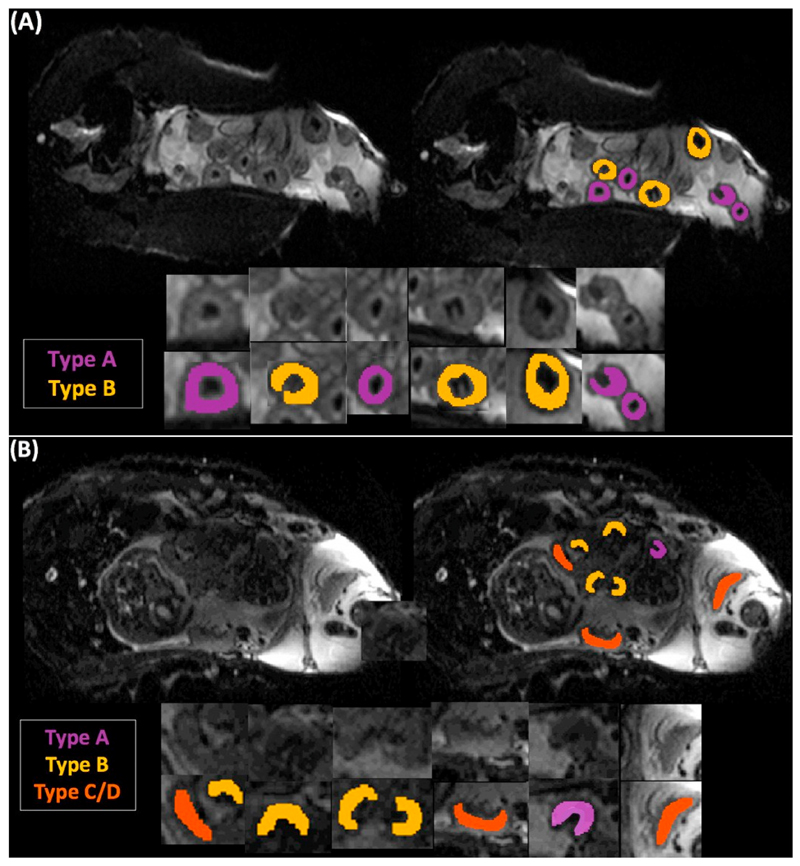
Example of MR images illustrating morphological classification of placentomes in a single sheep at 109–111 days GA (A) and 139–141 days GA (B).

Individual placentomes observed in 3D MRI scans were classified manually in a slice-by-slice manner based on morphology into three categories based on the classification system described by Ward et al. [38] at 109–111 days gestation (n = 14) and the 139–140 (n = 5) days gestation. Placentomes concave in shape with the maternal tissue (lighter grey-coloured) completely surrounding the fetal tissue (black) were classified as type A placentomes (Fig. 1A). Type B placentomes were categorised as those in which fetal tissue began to grow over the surrounding maternal tissue. Flat placentomes consisting of a large portion of fetal tissue that surrounds or has begun to surround maternal tissue were categorised as type C/D. Due to the comparatively low number of type C and D placentomes in control pregnancies, we chose to group these data (Fig. 1B). When placentomes were intermediate types, they were classified as the more advanced type, e.g. type B to type C/D was classified as type C/D. Placentome volume was calculated taking the number of voxels within each placentome and multiplying by the voxel dimensions.

### 2.7 Interrater reliability of MRI morphological classification

To investigate variability in placentome classification, placentomes were classified by two raters to achieve consensus. The raters were blinded to ewe information and gestational age (mid or late). Rater 1 (DF) has extensive experience working with MRI data, whereas Rater 2 (SLH) is an expert in post-mortem examinations. Rater 1 had specialist training on placentome morphology and also performed the classification of 3D MR images as described above. Since the manual segmentation of 3D images is a tedious and time-consuming task, the interrater reliability of the classification was performed on a subset of placentomes using a single 2D MR slice instead of the whole stack of MR slices. The 2D slice was selected to be in the centre of the approximately spherical placentome for best visibility. The interrater reliability test was performed on 122 placentomes.

### 2.8 Model fitting

The four different models were fitted voxel-by-voxel to the complete set of MRI measurements within the masked regions. We applied log-linear voxel-wise fitting to obtain measurements of ADC and T2 parameters using in-house software developed in MATLAB (The Math-Works, Natick, Massachusetts). To visualise directionality in DWI signal across b-values we also fit the DTI model (Eq. (3)). DTI data were processed using a custom written routine [48]. Parameter maps of FA were hence generated from DTI data as per standard calculations [48,50]. To investigate the effect of perfusion effects on the diffusion tensor fitting we also calculated FA values for b = 50 s.mm^−2^ and b = 100 s.mm^−2^. In addition, we calculated FA values by fitting data using both b = 50 s. mm^−2^ and b = 100 s.mm^−2^. IVIM analysis was performed using a Levenberg-Marquardt algorithm. IVIM-parameter perfusion fraction (*f*) was calculated with a voxel-wise bi-exponential fit according to Eq. (4). We constrained the parameters over a range of biologically plausible values: 0 < *f* < 1 (no units), 0 < ADC < 1 (mm^2^s^−1^), 0 < T2 < 500 (ms). These boundaries were the same across all ewes.

### 2.9 Statistical analysis

The statistical analysis was performed using R (version 3.3.2, 2017). Normality was assessed with the Shapiro-Wilk test. Two-sample *t*-test was performed to compare the MRI-derived placentome parameters across all animals between mid and late gestation. Paired *t*-test was also performed to compare the MRI placentome parameters between type A and type B placentomes at mid gestation. To analyse differences in MRI parameters between the three placentome type at late gestation, oneway ANOVA test was used. Numerical results are expressed as mean ± standard deviation (sd). Significance level was set at 5%.

## 3 Results

### 3.1 Placentome classification

**Fig. 2:**
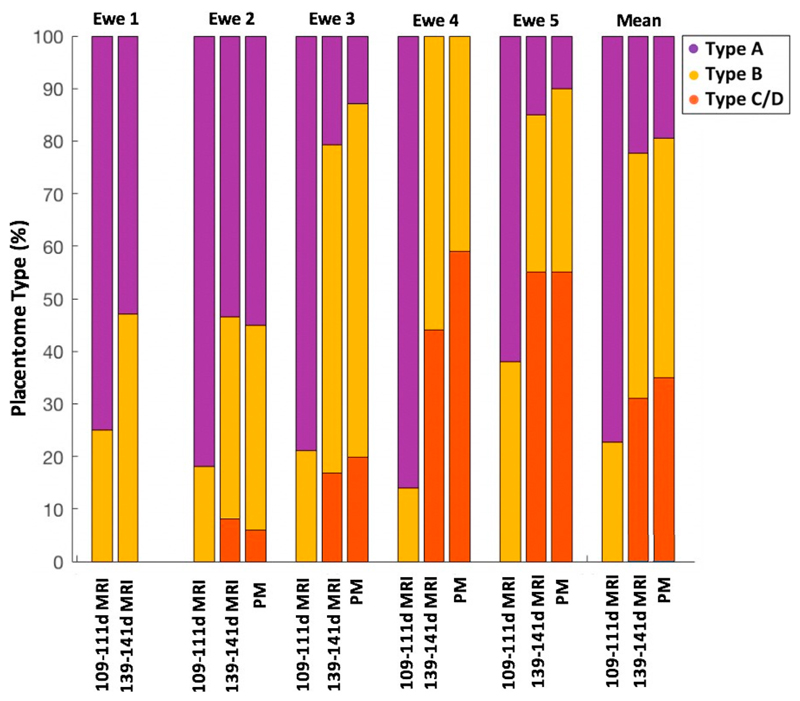
Morphological placentome classification results. Bar plots show the proportion of each placentome type. Each plot compares MRI classification results at mid and late gestational age with the post-mortem (PM) gold-standard data.

**Fig. 3:**
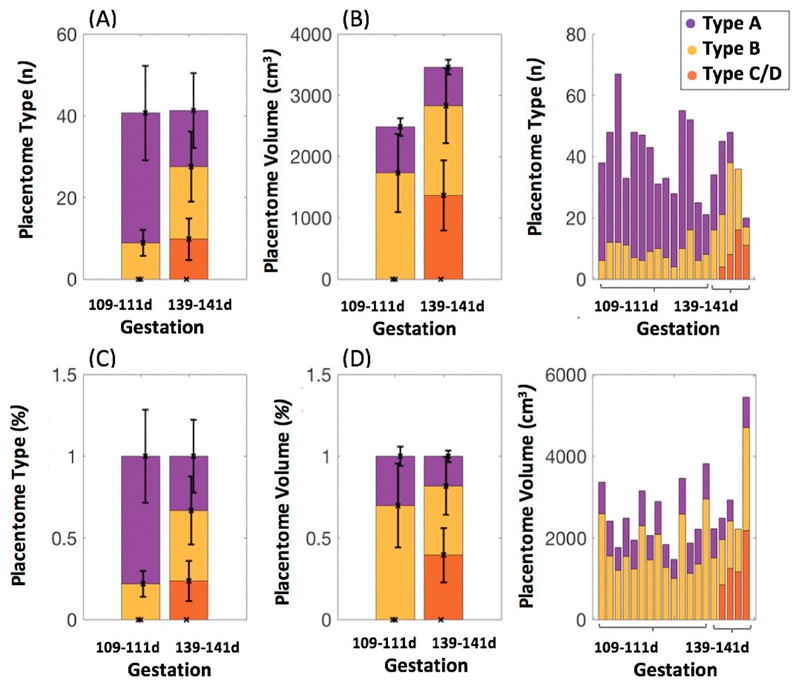
Histograms showing results estimated from MRI data at mid and late gestation: (**A**) total placentome number over all singleton pregnancies, (**B**) mean placentome volume, (**C**) total placentome number for each sheep, (**D**) percentage of placentome number over all singleton pregnancies, (**E**) percentage of placentome volume and (**F**) placentome volume for each sheep.

A total of 752 placentomes were visible on the MR images from all ewes included in the study. There was a longitudinal shift in the proportion of C/D type placentomes from 109–111 to 139–141 days of gestation (Table 1). We present the placentome total number, proportion and volume estimated from MRI data for each ewe at mid and late gestation in Supporting Tables S1 and S2. Fig. 2 visualises the accuracy in terms of agreement between placentome classification derived using MRI and the gold-standard post-mortem morphological approach. Proportions of each placentome type derived from MRI data in late gestation were similar to post-mortem data. Overall, 64% of post-mortem placentomes were segmented on MRI scans (Table S3). Classification of the placentome types using the MRI scans resulted in placentomes being categorised with 78.2% being type A and 21.8% being type B for ewes at 109–111 days of gestation. The scans at 139–141 days gestation resulted in proportions of 30.1% of type A, 48.6% of type B and 21.3% of type C/D. A decrease in volume of type A and type B was observed with age. Interestingly, no type C or D placentomes were identified at 109–111 days gestation. However, there was an increase in total placentome volume with increasing age associated with the increase in number of type C/D placentomes classified (Fig. 3). There was 87.7% overall agreement between the two raters. There was a small possibility of misclassification between type A and B of 4.9% and between type B and C/D of 7.4%. Disagreement in classification between type A and C/D placentomes was 0% (Fig. S1).

### 3.2 Quantitative MRI of the placentome

The average placentome estimates of ADC_109−111_ = 0.0016±5e^−4^ mm^2^s^−1^ and ADC_139−141_ = 0.0013±6e^−4^ mm^2^s^−1^ are consistent with cellular tissue in other vascular organs such as liver and human placenta (Table 2). There is a decrease in ADC with gestational age, which is most likely explained by the low ADC of type C/D (Table 2). There is also a decrease in T2 with gestational age. The average T2 values were T2_109−111_ = 152.2 ± 58.1 ms and T2_139−141_ = 127.8 ± 52.0 ms, which are consistent with a highly perfused and oxygen saturated tissue. FA was significantly higher at the low b-value (FA_109−111(b=50)_ = 0.762 ± 0.22, FA_139−141(b=50)_ = 0.849 ± 0.21) compared to the high b-value (FA_109–111(b=100)_ = 0.53 5 ± 0.22, FA_139−141(b=100)_ = 0.691 ± 0.25), suggesting that is related to the radial blood flow component of the sheep placentome. An increase with gestation is observed in the average placentome estimates of *f*_109−111_ = 0.22 ± 0.12 and f_139−141_ = 0.29 ± 0.16 (no units) (Fig. S2).

**Fig. 4:**
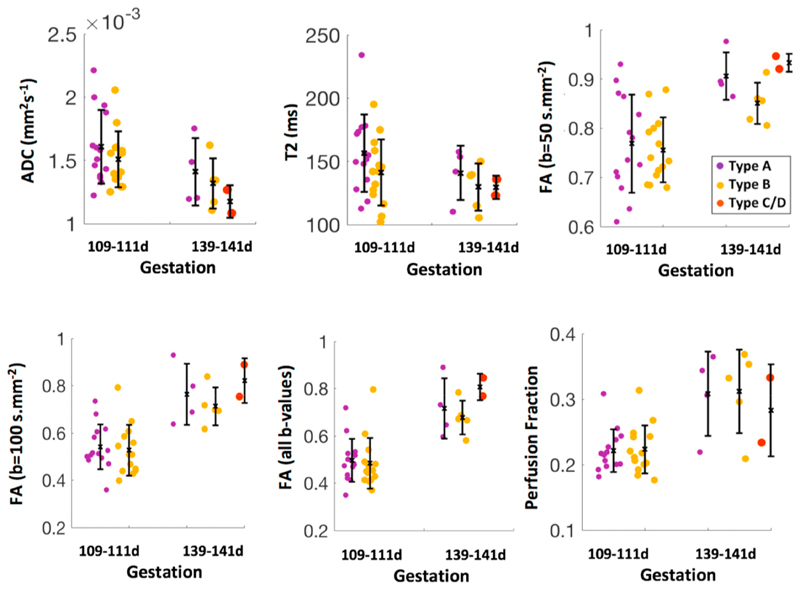
Boxplots summarising results of changes in MRI parameters with stage of gestation for each placentome type in all singleton pregnancies. Each plot shows the median as well as the 25th and 75th percentile.

Fig. 4 shows the MRI parameter estimates for the three placentome types at 109–111 and 139–141-days gestation. There were significant differences between type A and type B placentome for ADC_109−111_ and T2_109−111_ (P = 0.037, 0.006). However, there was no evidence of a significant difference across type A and type B for FA_109−111(b=50)_, FA_109–111(b=100_, FA_109–111(all b-values)_ and *f*_109–111_. No significant difference was observed between the three placentome types at 139–141 days gestation.

## 4 Discussion

In this study, we applied quantitative MRI imaging to *in-vivo* sheep placental tissue. Our results show consistent patterns with known gestational age changes in sheep placental development, as supported by post-delivery gold standard placentome examination.

We demonstrated that placentome morphology changes in the same animals across gestation. This is consistent with the results of crosssectional analysis in the literature [38]. Using the MRI data, we were able to segment just under two-thirds of the placentomes that were found at post-mortem. The discrepancy was mainly due to limitations in the field of view for the diffusion acquisition. We showed that there are morphological changes in shape and structure between ~ 110 and ~ 140 days gestation and that this was consistent with post-delivery classification proportions. The reduction in proportion and average volume of type A placentomes likely reflects the conversion of placentomes progressively from type A to type B or C/D, rather than an atrophy of type A placentomes. Inter-rater variability indicated that there is some possibility of inconsistent classification between different placentome types. Discrimination of type A vs B and type B vs C/D was most likely to be disparate between raters. No disagreement was observed between type A and type C/D placentomes.

Observations of ADC estimates showed a decrease with gestational age agreeing with previous studies in the human placenta [51]. There was a decrease in T2 of 24.4 ms with increasing gestation, which is like effects seen in human pregnancy [20,22,24,52]. It is reasonable to expect that fetal blood oxygen saturation and T2 will fall slightly with increasing gestation in the fetal sheep [63], whilst maternal blood will maintain a similar T2 value throughout pregnancy [54]. A significantly higher diffusion anisotropy was observed at b-value = 50 s.mm^−2^, suggesting that the perfusion component plays a role in the observation of placental tissue anisotropy. In the sheep, the exchange takes place between interdigitated fetal and maternal villi, giving rise to a directional structure as inferred from the high FA at low b-value. In the human placenta, there is no maternal villus structure, and the villus tree is free floating in the maternal blood pool and thus this is likely to be a species-specific observation. Perfusion fraction values showed an increase with gestational age, which is in keeping with the increased blood delivery with gestational age that is common between human and sheep placenta. As a measurement of vascular density, these values are low in comparison to human placenta but comparable to other vascular organs such as the human liver [55].

Our results suggest some evidence for increasing blood flow during gestation. This interpretation is in line with the placentome anatomy, which consists of maternal (caruncular) and fetal (cotyledonary) vascular systems. Based on previous literature [39], the architecture of the caruncle remains similar throughout pregnancy, but the thickness of the caruncle increases due to increased vascularity in late gestation. Similarly, cotyledon architecture remains similar during pregnancy, but branching of the cotyledonary vessels increases later in gestation. It has also been reported that areas of the distal fetal villi align with maternal villi with mixed counter-current and cross-current blood flow of the maternal and fetal sides. Thus, the increase of FA and *f* in late gestation is consistent with these findings [39,56].

Preclinical rodent models have shown the utility of MRI for measuring flow and function in a small labyrinthine placenta [57,58], whilst results in non-human primates are relevant due to their invasive placentation [16,59–61]. However, within mammalian orders there is significant diversity of placentation, each of these models have their own statements to make about placentation, but each have their own limitations when applied to inform on human pregnancy. Sheep are a large animal model of human pregnancy with the advantage of being amenable to surgical intervention, although with their own distinct placental type.

This study has several limitations. First, the small number of animals studied particularly at 139–141 days gestation. Moreover, the experimental setting with surgery and anaesthesia may have affected our results due to cardio-respiratory depression [45,53]. However, anaesthesia is required to prevent the ewe from being distressed and to limit ewe and fetal movement [62]. Placentome volume estimates are generally lower than those previously reported [47]. This is likely because placentome ROIs were drawn fully within the boundary of placentomes to minimise the impact of noise from movement impacting the analysis. It is also possible that some placentomes were not clearly visible on some MRI slices, and thus could not be analysed. Movement artefact is a potential issue and can lead to poor quality images. A non-rigid motion correction was applied prior to the analysis of MRI data to correct for motion. However, it is possible that residual errors from partial volume effects may have played a role in the analysis. Misclassification may arise when placentomes are on the borderline between type A and B or between type B and C. Placentomes of type A and B appear similar in shape, but type B placentomes consist of fetal tissue beginning to grow over the surrounding maternal tissue. However, this may not be clearly visible on MRI. Misclassification may also arise due to rater experience and expertise, although here we find good inter-rater agreement.

This work has provided baseline data for placental MRI techniques in a relevant animal model of placental function. We characterised the diffusion and relaxation properties of sheep placentomes in uncomplicated pregnancy. This study provides findings that will help support the future translation of advanced MR measurements and placental medicine into the clinical setting.

## Supplementary Material

Supplementary data to this article can be found online at https://doi.org/10.1016/j.placenta.2021.01.017.

Supplementary data

## Figures and Tables

**Table 1:** Summary of placentome total number, proportion and average volume for each category estimated from MRI for all pregnancies.

Placentomes	Total Number		Proportion (%)		Average Volume (cm^3^)	
	109-111 d	139-141 d	109-111 d	139-141 d	109-111 d	139-141 d
Type A	445	55	78.2	30.1	2432	2040
Type B	124	89	21.8	48.6	5607	4755
Type C/D	0	39	0	21.3	0	6226

**Table 2:** Left-to-Right: Average MRI parameters derived over all singleton pregnancies at two gestational ages for all placentomes. Two-sample *t*-test was performed to compare the MRI-derived parameters. Significant differences are (P < 0.05) are shown in bold. Average MRI parameters of the three placentome types at two gestational ages. Results are presented as mean± standard deviation (sd).

MRI Parameter	All Placentomes Mean ± sd		Type A		Type B		Type C/D	
	109-111d	139-141d	109-111d	139-141d	109-111d	139-141d	109-111d	139-141d
ADC (mm^2^s^−1^	0.0016 ± 5e^−4^	**0.0013 ± 6e^4^**	0.0017 ± 5e^−4^	0.0015 ± 5e^−4^	0.0015 ± 4e^−4^	0.0013 ± 5e^−4^	0	0.0012 ± 5e^−4^
T2 (ms)	152.2 ± 58.1	**127.8 ± 52.0**	158.5 ± 48.7	141.1 ± 70.4	141.3 ± 58.5	130.3 ± 53.3	0	130.0 ± 55.2
FA (b = 50 s.mm^−2^)	0.762 ± 0.22	**0.849 ± 0.21**	0.769 ± 0.23	**0.883 ± 0.22**	0.756 ± 0.23	**0.851 ± 0.19**	0	0.902 ± 0.17
FA (b = 100 s.mm^-2^)	0.535 ± 0.22	**0.691 ± 0.25**	0.542 ± 0.23	**0.708 ± 0.25**	0.527 ± 0.21	**0.713 ± 0.25**	0	0.780 ± 0.23
FA (all b-values)	0.479 ± 0.21	**0.658 ± 0.26**	0.496 ± 0.22	**0.665 ± 0.26**	0.484 ± 0.19	**0.684 ± 0.25**	0	0.749 ± 0.23
***f*** (no units)	0.223 ± 0.12	**0.291 ± 0.16**	0.224 ± 0.22	**0.306 ± 0.30**	0.233 ± 0.11	**0.315 ± 0.13**	0	0.258 ± 0.16
