# Supplementary material for: Magnetic resonance imaging of placentome development in the pregnant Ewe
Source: Placenta. Author manuscript; Available in PMC 2021 Aug 1. (PMC7611430; doi:10.1016/j.placenta.2021.01.017)
Supplement: Supplementary data [file EMS131075-supplement-Supplementary_data.zip › 1-s2.0-S0143400421000254-mmc1.pdf]

Table S1
Summary of placentome total number, proportion and average volume estimated from MRI data for each ewe at 109-111 days gestation. At mid gestational age Type C/D placentomes were not observed.

Ewe Nº	Nº of placentomes		Proportion (%)		Volume (cm^3)	
	Type A	Type B	Type A	Type B	Type A	Type B
**1**	32	6	84.2	15.8	2518	8375
**2**	36	12	75	25	2777	5041
**3**	55	12	82.1	17.9	1837	3888
**4**	22	11	66.6	33.3	3042	4993
**5**	41	7	85.4	14.6	2287	4014
**6**	41	6	87.2	12.8	2786	7420
**7**	34	9	79.1	20.9	1934	4714
**8**	21	10	67.7	32.3	2644	6748
**9**	26	7	78.8	21.2	1844	4115
**10**	24	4	85.7	14.3	1526	3250
**11**	45	10	81.8	18.2	2854	8349
**12**	36	16	69.2	30.8	2417	3658
**13**	19	6	76	24	2748	4397
**14**	13	8	61.9	38.1	2832	9542
