# Supplementary material for: Magnetic resonance imaging of placentome development in the pregnant Ewe
Source: Placenta. Author manuscript; Available in PMC 2021 Aug 1. (PMC7611430; doi:10.1016/j.placenta.2021.01.017)
Supplement: Supplementary data [file EMS131075-supplement-Supplementary_data.zip › 1-s2.0-S0143400421000254-mmc3.pdf]

Table S3

Characteristics of the late GA study population. Placental efficiency is denoted as fetal weight/placental weight (dimensionless).

Animal	Fetal Weight (kg)	Placental Weight (g)	Placental Efficiency	Post-mortem (PM) Placentome Number	MRI Placentome Number	MRI-derived Placentomes Compared to PM (%)
Ewe 1	4.945	360.930	13.7	64	45	70.3
Ewe 2	4.325	385.551	11.2	77	48	62.3
Ewe 3	4.935	384.512	12.8	63	37	58.7
Ewe 4	4.610	360.619	12.8	31	20	64.5
