# Supplementary material for: Magnetic resonance imaging of placentome development in the pregnant Ewe
Source: Placenta. Author manuscript; Available in PMC 2021 Aug 1. (PMC7611430; doi:10.1016/j.placenta.2021.01.017)
Supplement: Supplementary data [file EMS131075-supplement-Supplementary_data.zip › 1-s2.0-S0143400421000254-mmc2.pdf]

Table S2

Summary of placentome total number, proportion and average volume estimated from MRI data for each ewe at 139-141 days gestation.

Ewe Nº	Nº of placentomes			Proportion (%)			Volume (cm^3)		
	Type A	Type B	Type C/D	Type A	Type B	Type C/D	Type A	Type B	Type C/D
**1**	18	16	0	52.9	47.1	0	2343	4879	0
**2**	24	17	4	53.3	37.7	9	1711	3596	4574
**3**	10	30	8	20.8	62.5	16.7	1695	3732	4069
**4**	0	20	16	0	55.6	44.4	0	3392	4797
**5**	3	6	11	15	30	55	2398	8168	11471
