# Supplementary material for: Magnetic resonance imaging of placentome development in the pregnant Ewe
Source: Placenta. Author manuscript; Available in PMC 2021 Aug 1. (PMC7611430; doi:10.1016/j.placenta.2021.01.017)
Supplement: Supplementary data [file EMS131075-supplement-Supplementary_data.zip › 1-s2.0-S0143400421000254-mmc4.pdf]

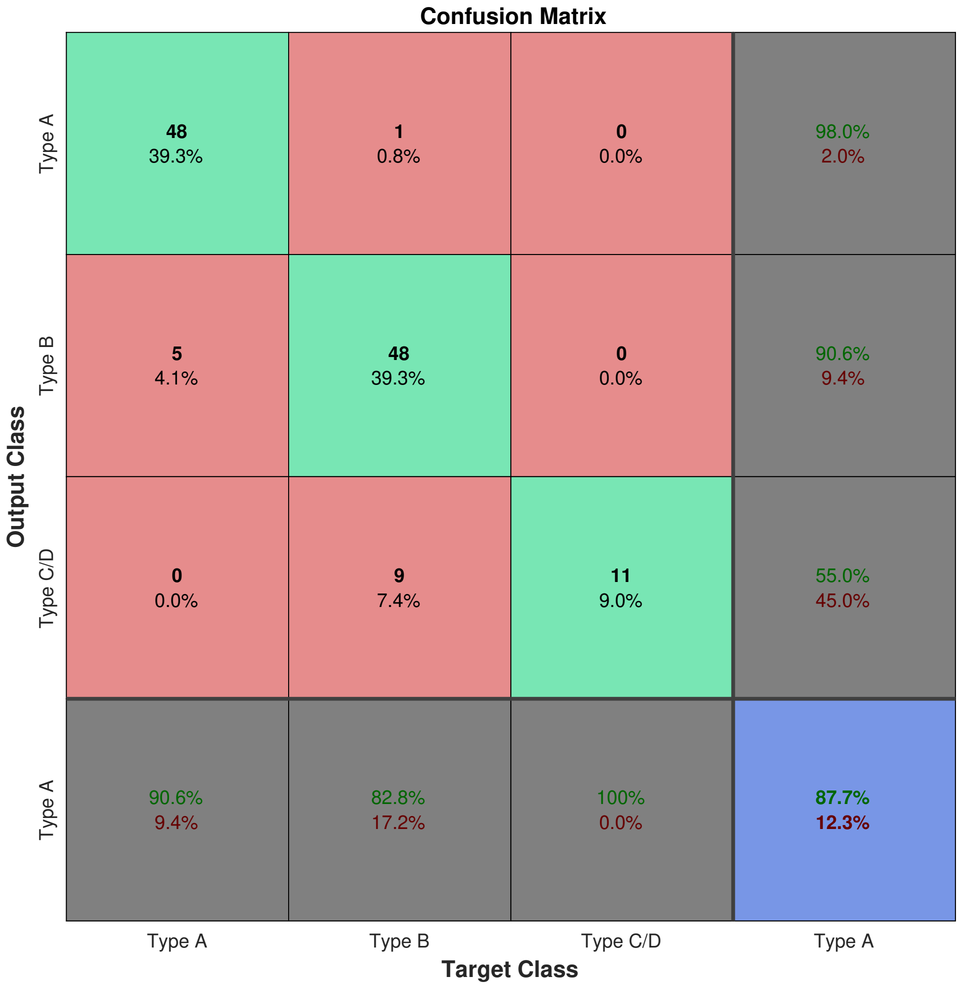

Figure S1: Confusion matrix for classification results rated by two operators using a subset of placentomes derived from 9 ewes. The diagonal cells (green) show the number and percentage of correct classification by the two raters. The off-diagonal cells (red) correspond to incorrectly classified observations. The column on the far right of the plot and the bottom row of the plot (grey cells) show the percentages of all the placentomes predicted to belong to each type that are correctly and incorrectly classified. The blue cell in the bottom right shows the overall agreement.
