# Supplementary material for: Magnetic resonance imaging of placentome development in the pregnant Ewe
Source: Placenta. Author manuscript; Available in PMC 2021 Aug 1. (PMC7611430; doi:10.1016/j.placenta.2021.01.017)
Supplement: Supplementary data [file EMS131075-supplement-Supplementary_data.zip › 1-s2.0-S0143400421000254-mmc5.pdf]

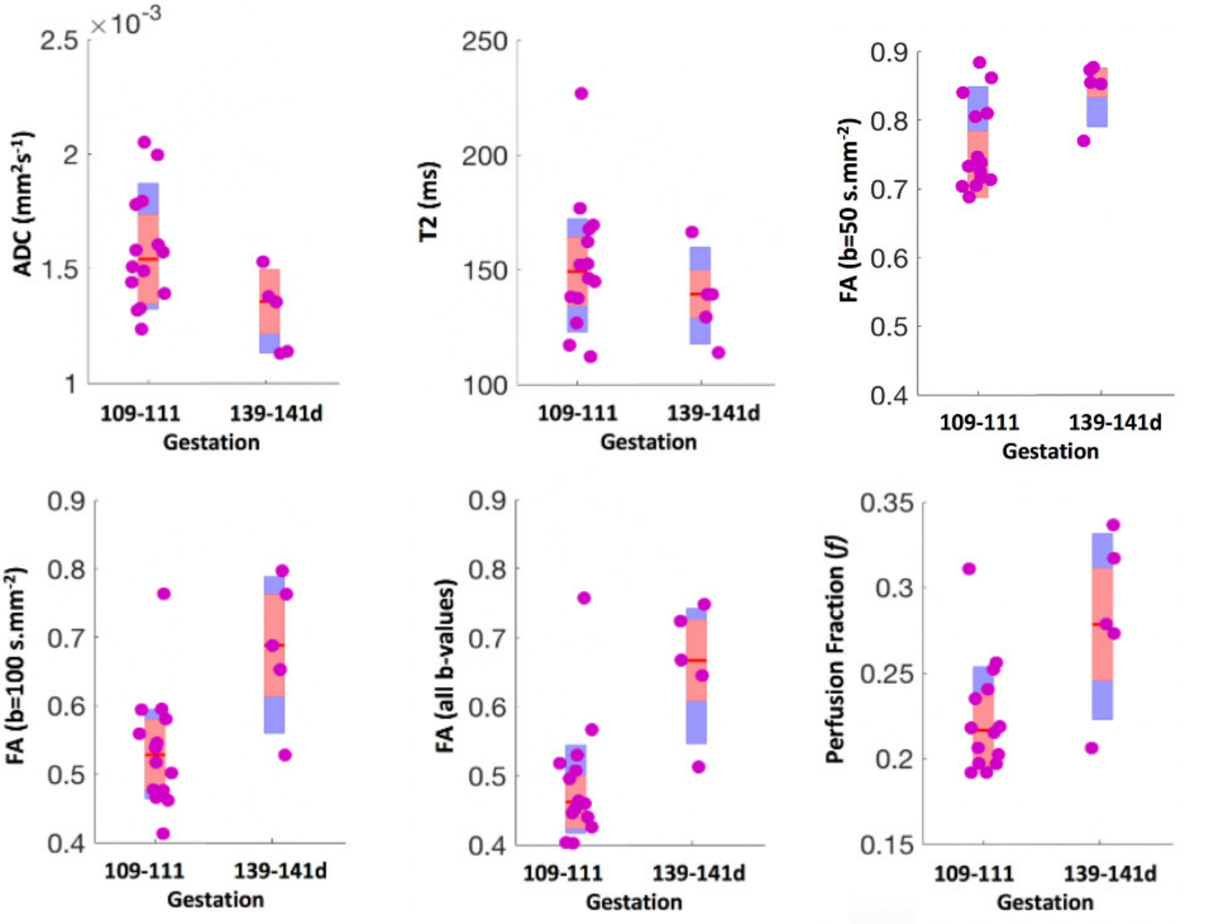

Figure S2: Boxplots summarising results over all singleton pregnancies at mid (n=14) and late gestation (n=5). Each plot shows: the median (red line), the 25th and 75th percentile (purple box) and individual means of each sheep (pink circles).
